# Lipoprotein Particle Profiles Mark Familial and Sporadic Human Longevity

**DOI:** 10.1371/journal.pmed.0030495

**Published:** 2006-12-26

**Authors:** Bastiaan T Heijmans, Marian Beekman, Jeanine J Houwing-Duistermaat, Mark R Cobain, Jonathan Powell, Gerard Jan Blauw, Frans van der Ouderaa, Rudi G. J Westendorp, P. Eline Slagboom

**Affiliations:** 1 Department of Molecular Epidemiology, Leiden University Medical Centre, Leiden, The Netherlands; 2 Department of Medical Statistics, Leiden University Medical Centre, Leiden, The Netherlands; 3 Unilever Corporate Research, Colworth Laboratory, Sharnbrook, Bedford, United Kingdom; 4 Department of Gerontology and Geriatrics, Leiden University Medical Centre, Leiden, The Netherlands; University of Newcastle upon Tyne, United Kingdom

## Abstract

**Background:**

Genetic and biochemical studies have indicated an important role for lipid metabolism in human longevity. Ashkenazi Jewish centenarians and their offspring have large low-density lipoprotein (LDL) and high-density lipoprotein (HDL) particles as compared with control individuals. This profile also coincided with a lower prevalence of disease. Here, we investigate whether this observation can be confirmed for familial longevity in an outbred European population and whether it can be extended to sporadic longevity in the general population.

**Methods and Findings:**

NMR-measured lipoprotein profiles were analyzed in 165 families from the Leiden Longevity Study, consisting of 340 long-lived siblings (females >91 y, males >89 y), 511 of their offspring, and 243 partners of the offspring. Offspring had larger (21.3 versus 21.1 nm; *p* = 0.020) and fewer (1,470 versus 1,561 nmol/l; *p* = 0.011) LDL particles than their same-aged partners. This effect was even more prominent in the long-lived siblings (*p* < 10^−3^) and could be pinpointed to a reduction specifically in the concentration of small LDL particles. No differences were observed for HDL particle phenotypes. The mean LDL particle sizes in 259 90-y-old singletons from a population-based study were similar to those in the long-lived siblings and thus significantly larger than in partners of the offspring, suggesting that the relevance of this phenotype extends beyond familial longevity. A low concentration of small LDL particles was associated with better overall health among both long-lived siblings (*p* = 0.003) and 90-y-old singletons (*p* = 0.007).

**Conclusions:**

Our study indicates that LDL particle profiles mark both familial and sporadic human longevity already in middle age.

## Abstract 2

Offspring of families from the Leiden Longevity Study had larger and fewer LDL particles than same-aged partners, suggesting that even in middle age LDL particle profiles are associated with longevity.

## Editors' Summary

**Background.:**

It is not clear why some people go on to live longer than others do. Some studies have shown that close relatives of long-lived people are themselves more likely to live for a long time; these findings suggest that there is probably a genetic basis for long life. However, the actual mechanisms involved have not yet been worked out. Some genes coding for proteins involved in fat metabolism, such as *APOE, APOB,* and *CETP,* have been associated with long life, suggesting a link between the way fat gets metabolized and the aging process. One study that supports this idea found that the children of 100-year-old people had larger lipoprotein particles (assemblies of proteins and fats that carry cholesterol and triglycerides in the blood) than similarly aged control individuals. However, studies such as this are very prone to “false positive” findings and therefore need to be backed up by confirmatory evidence. In addition, the previous study was performed in a very specific population (Ashkenazi Jewish people), and it is important to find out whether the findings are also true in other populations.

**Why Was This Study Done?:**

The research group carrying out this study wanted to address several distinct questions to do with the genetics of aging. Firstly, they wanted to see if they could confirm previous findings associating large lipoprotein particles with longer life, but looking at people who were more representative of the general European population and not from a genetically isolated population. Secondly, they wanted to see whether this association applied to only long-lived people whose family members were also long-lived, or to long-lived people in general. Finally, they wanted to find out if the large lipoprotein particles were associated with better health.

**What Did the Researchers Do and Find?:**

In the study, the researchers looked at long-lived people from across The Netherlands whose relatives were also long-lived. For this, they recruited 340 men aged over 89 and women aged over 91 into the study, all of whom had at least one similarly long-lived sister or brother. Their children (511 individuals), and the partners of their children (243 people), were also recruited into the study, with the partners acting as “controls.” The researchers also studied 259 people who had just turned 90 years old; these people were included to see whether particular characteristics of lipoproteins existed in long-lived people whose longevity did not run in families. All the participants gave blood samples, and the researchers then measured the size and amount of different lipoprotein particles in these samples. Two types of lipoprotein particles were looked at: low-density lipoprotein (LDL, often termed “bad cholesterol”) and high-density lipoprotein (HDL, sometimes called “good cholesterol”). The researchers found that the children from the long-lived people had larger and fewer LDL particles than their partners (the “control” individuals) just like their long-lived parents. Thus even though the children were not long-lived themselves, LDL particles marked the fact that they have a higher chance of becoming long-lived in the future. Similar changes in LDL particles were found for long-lived people whose relatives were not also long-lived. Interestingly, simply the level of cholesterol—the classical risk factor for cardiovascular disease—did not appear to play a role. Thus it seems that it is not the amount of cholesterol that is important in longevity but how it is packaged. Better health status was also associated with a lower proportion of small LDL particles in the blood, supporting these findings. No characteristics of the HDL particles seemed to be associated with longevity.

**What Do These Findings Mean?:**

These findings confirm those from a previous study in Ashkenazi Jewish people that suggested that the size of LDL particles in the blood was associated with long life. The nature of this association is not clear; some studies indicated that small LDL particles increase the risk of cardiovascular disease but small LDL particles may also be harmless themselves and reflect the efficiency of other processes causally related to aging.

**Additional Information.:**

Please access these Web sites via the online version of this summary at http://dx.doi.org/10.1371/journal.pmed.0030495.

- Wikipedia chapter on senescence (biology of aging) (note that Wikipedia is a free Internet encyclopedia that anyone can edit)
- US National Institute on Aging provides information on healthy aging, details of publicly funded research into aging, and other resources for the public
- Help the Aged information on research into aging

## Introduction

The study of families displaying exceptional longevity may reveal the biological basis for a healthy ageing process [1,2]. First-degree relatives of centenarians [3] and nonagenarian siblings [4] have a lifelong mortality advantage as compared with their birth cohort. These findings imply that there is a substantial familial, possibly genetic, component to exceptional longevity. Moreover, they indicate that the factors involved in longevity express their beneficial effect at earlier ages and confer resistance to multiple common diseases. Indeed, the offspring of centenarians with a mean age of 71 y have a decreased prevalence of [5] and mortality from [6] coronary heart disease, diabetes, and cancer. Studying genetic and biochemical markers in long-lived individuals and their offspring may therefore reveal biological mechanisms that protect against a broad range of common diseases perhaps secondary to a decreased rate of biological ageing [5].

Human population studies have indicated a significant role for lipid metabolism in the survival to exceptionally high ages. Variants of a number of genes involved in this pathway, including *APOE* [2], *APOB* [7] and *CETP* [8], were associated with human longevity. The most compelling indication for a role of lipid metabolism came from the study of NMR-measured subclasses of lipoprotein particles in long-lived Ashkenazi Jewish families. Offspring of centenarians had strikingly larger low-density lipoprotein (LDL) and high-density lipoprotein (HDL) particles than age-matched controls, and even larger particles were observed among the centenarians themselves [8]. Larger LDL and HDL particles were further associated with lower prevalence of hypertension, cardiovascular disease, and the metabolic syndrome in offspring of centenarians and their spouses [8].

To definitively establish the relevance of lipoprotein particle profiles to human longevity, we set out to answer four questions. The first question was whether the association observed in the Ashkenazi Jewish founder population could be confirmed in and broadened to an outbred European population. The second was whether the beneficial lipoprotein profiles marked only familial exceptional longevity or also extended to sporadic longevity in the general population. The third question was whether the observed associations depended on triglyceride levels, a major determinant of lipoprotein particle size [9,10]. And finally, we assessed whether beneficial protein profiles were also associated with a better health status.

To answer these questions, we studied lipoprotein particle profiles measured using NMR in two populations. We investigated familial longevity in long-lived siblings, their offspring, and the partners of their offspring (Leiden Longevity Study) [4]. Sporadic longevity reflecting the phenotype in the general population was studied in 90-y-old participants from a population-based study (the Leiden 85-plus Study) [11].

## Methods

### Participants

#### Leiden Longevity Study.

For the Leiden Longevity Study [4], long-lived siblings were recruited together with their offspring and the partners of their offspring. Families were eligible for the study if (i) at least two long-lived, full siblings were alive, where men were considered to be long-lived if they were 89 y or older and women if they were 91 y or older, and (ii) the parents of the sibship were from Dutch descent. In 2001, less than 0.5% of the Dutch population fulfilled these sex-specific age criteria. Ages were verified using passport, birth certificate, or marriage certificate. The study population is enriched for familial influences on longevity because offspring of the long-lived siblings, as well as other first-degree relatives, have an on-average 30% lower mortality rate as compared with their birth cohort [4]. In the long-lived siblings, overall health status was estimated using questionnaires on (instrumental) activities of daily living (ADL) [12,13]. In case of cognitive impairment, these data were obtained from a guardian.

#### Leiden 85-plus Study.

Between September 1997 and September 1999, all inhabitants of Leiden were asked to participate in the Leiden 85-plus Study within a week after they turned 85 y, irrespective of health status [11]. As part of the follow-up of the study, all participants were visited again when they reached 90 y. The 90-y-old participants from this population (90-y-old singletons) were considered sporadic nonagenarians as opposed to the nonagenarian siblings in the Leiden Longevity Study selected for their familial longevity background. During the interview with participants, competence in ADL was measured with the Groningen Activity Restriction Scale (GARS) [14]. For participants with severe cognitive impairment, information was obtained from a responsible person. Both the Leiden 85-plus Study and the Leiden Longevity Study were approved by the ethical committee of the Leiden University Medical Centre, and all participants signed an informed consent.

### Classical Lipids and NMR Analysis of Lipoprotein Subclasses

A venous blood sample was drawn from the participants. Classical lipids, including total cholesterol, HDL cholesterol, and triglycerides, were measured using standard methods. LDL cholesterol level (LDL-C) was calculated using the Friedewald formula (LDL-C = total cholesterol − HDL-C − (triglycerides/2.2); unit mmol/l) and set to missing if plasma triglyceride concentration exceeded 4.52 mmol/l. For lipoprotein particle measurement, serum was available in the Leiden Longevity Study and citrate plasma in the Leiden 85-plus study. Vacuum tubes for plasma collection contained citrate in liquid form leading to dilution and osmotic effects. Therefore, only mean lipoprotein particle sizes, which are not affected by these effects, but not particle concentrations could be used for comparisons across the two study populations. The NMR lipoprotein subclass profile was determined using a 400-MHz proton NMR analyzer at LipoScience (http://www.liposcience.com). In brief, the NMR method uses the characteristic signals broadcast by lipoprotein subclasses of different size as the basis of their quantification [15]. Each measurement produces the signal amplitudes of lipoprotein subclasses that allows one to estimate the total LDL and HDL particle concentration as well as their subclasses including large (21.3–23.0 nm) and small (18.0–21.2 nm) LDL particles. For this estimation, conversion factors are used to relate these signal amplitudes to particle concentration or lipid mass concentration units, which were obtained from NMR and chemical lipid analyses of a set of purified subclass standards. Particle concentrations (nmol/l) were derived for each subclass standard by measuring the total concentration of core lipid (cholesterol ester plus triglyceride) and dividing the volume occupied by these lipids by the core volume per particle calculated from knowledge of the particle's diameter. Triglyceride concentrations were calculated from NMR subclass measurements. Average particle sizes (diameter in nanometres) of LDL and HDL were determined by weighting the relative mass percentage of each subclass according to its diameter. Note that due to the relatively small range in diameters for various LDL and HDL lipoprotein subclasses and the weighting, limited absolute inter-individual differences exist in mean particle sizes (resulting in small standard deviations and standard errors). NMR LDL sizes are consistent with electron microscopy data and uniformly ~5 nm smaller than those estimated by gradient gel electrophoresis due to different referencing.

### Statistical Analysis

Mean particle sizes were normally distributed. To obtain normally distributed variables for particle concentrations, a square-root transformation was used. Back-transformed data are presented, and standard errors were calculated using the delta method. Differences in particle sizes and concentrations for LDL and HDL particles between long-lived siblings, their offspring, and the partners of their offspring were assessed using linear regression. In these analyses, the three groups of individuals were included as a categorical variable together with age and sex. To take into account dependencies within families (i.e., a long-lived sibship with all offspring and their partners), robust standard errors were used, i.e., the variance was computed from the between-family variation [16]. *p*-Values were also based on these robust standard errors. To assess the robustness of our findings, we also performed the analyses in the subset of complete partner offspring pairs. Furthermore, interaction with sex was tested.

Mean particle sizes were also compared between 259 90-y-old singletons and the partners, offspring, and long-lived siblings using linear regression. Associations were again adjusted for age and sex, and the within-family correlation was taken into account when computing standard errors. To study whether the identified relationships between particle phenotypes and longevity depended on triglyceride levels, we repeated all analyses including triglyceride levels as covariate in the model. In the analysis of the association with competence measures in ADL, a measure of overall health in the very old, the ADL score was used as a continuous variable. All analyses were performed using STATA SE version 8 (http://www.stata.com).

## Results

**Figure 1:**
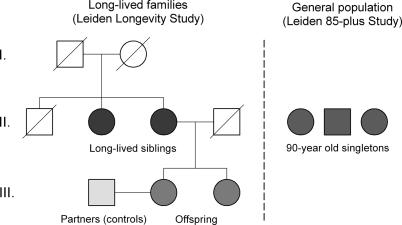
Study Design Long-lived families were recruited if they included a long-lived sibling pair with males aged 89 y or older and women aged 91 y or older.

To test lipoprotein profiles for their association with longevity, we recruited 1,094 individuals from 165 families consisting of long-lived siblings (probands; *n* = 340), their offspring (*n* = 511), and partners of their offspring (*n* = 243; Figure 1). To assess the relevance of our findings for sporadic longevity (longevity not enriched for familial influences), we studied the lipoprotein profiles in 90-y-old singletons from the general population (*n* = 259; Figure 1).

Classical lipid levels as well as the mean size and concentration of lipoprotein particles were measured in partners, offspring, and long-lived siblings by NMR. Offspring had larger (*p* = 0.020) and fewer (*p* = 0.011) LDL particles than their partners (Table 1). These differences were present at all ages (Figure S1) and more apparent when partners were compared with long-lived siblings (both *p* < 10^−3^). This finding is in agreement with the expectation that factors related to the familial longevity phenotype are diluted in the offspring because only one of their parents was selected for longevity. In contrast, the mean HDL particle size was virtually identical in offspring and partners and the HDL particle concentration not significantly lower in offspring than in partners (Table 1). Larger HDL particles and lower particle concentration were found among long-lived siblings. For classical HDL-cholesterol and triglyceride levels, no significant difference was observed between partners and offspring. Offspring did, however, have significantly higher LDL-cholesterol levels than their partners (Table 1). Such higher LDL-cholesterol levels were not observed among long-lived siblings.

**Table 1:**
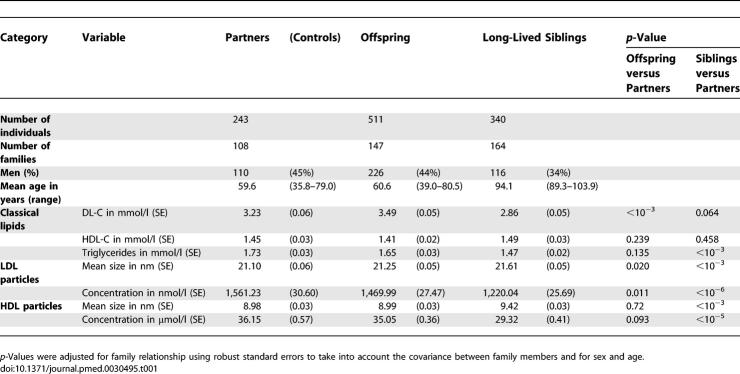
Characteristics, Classical Lipids, and LDL and HDL Particle Size and Concentration in Individuals with an Increasing Familial Propensity for Longevity

To assess the robustness of these findings, we tested for differences in lipoprotein particle profiles within partner–offspring pairs (236 complete pairs available). The age- and sex-adjusted LDL particle size was 0.11 nm larger (*p* = 0.079) and the LDL particle concentration 109 nmol/l lower (*p* = 0.009) in offspring than in their partners. No differences for HDL particle phenotypes were observed (*p* = 0.67 and *p* = 0.18 for particle size and concentration, respectively).

**Figure 2:**
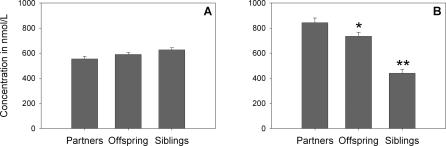
Concentration of Large and Small LDL Particles in Serum of Offspring and Long-Lived Siblings Compared with Partners (A) Large LDL particles; (B) small LDL particles. * *p* ≤ 0.02 for comparison versus partners (controls); ** *p* ≤ 10^−3^ for comparison versus partners (controls).

A closer inspection of the association found for LDL particles showed that it could be attributed to a 13% lower small LDL particle concentration (*p* = 0.012) in offspring as compared with partners but not a higher concentration of large LDL particles (*p* = 0.19). Again, the contrast was even greater in long-lived siblings who had on average a 48% lower concentration of small LDL particles than partners of their offspring (*p* < 10^−4^; Figure 2). Subclassification of the total HDL particle concentration according to size did not reveal consistent significant associations (unpublished data).

Next, we investigated men and women separately. Among men, the association between familial longevity and total LDL particle concentration was highly significant. Offspring had a 12% (*p* = 0.001) and long-lived siblings a 30% (*p* = 10^−4^) lower particle concentration than partners (Table 2). Although the same trend was observed among women, this was significantly attenuated (*p*_interaction_ = 0.043). As a consequence, the 15% higher LDL particle concentration in men than women among partners was inverted to 5% lower concentration in men than women among partners among long-lived siblings. Separate analysis of men and women further suggested that, if present, the association of total HDL particle concentration with a familial propensity for longevity was mainly found among women (*p* = 0.037 for the comparison of partners with offspring).

**Table 2:**
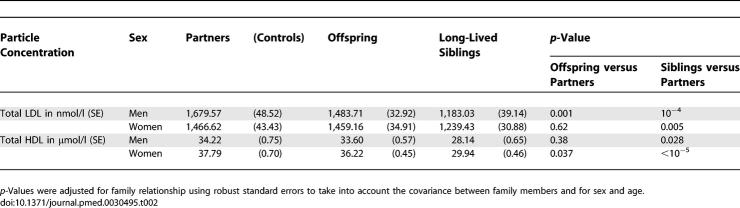
Total LDL and HDL Particle Concentration Separately for Men and Women with an Increasing Familial Propensity for Longevity

**Figure 3:**
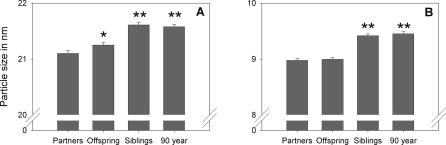
Mean Particle Sizes of LDL and HDL in Individuals from Long-Lived Families and 90-y-Old Singletons from the General Population (A) LDL; (B) HDL. * *p* ≤ 0.02 for comparison versus partners (controls); ** *p* ≤ 10^−3^ for comparison versus partners (controls).

To test whether the relevance of lipoprotein particle subclasses extended to the general population, we measured LDL and HDL particle profiles in 259 90-y-old singletons. Since a different sample type was available (now plasma instead of serum), only mean particle sizes could be used for comparison with long-lived siblings. The mean LDL particle size in 90-y-old singletons was similar to that in long-lived siblings (21.58 nm and 21.61 nm, respectively) and also significantly larger than in partners of offspring (*p* = 0.001; Figure 3). For HDL particles, the same observation was made: mean sizes were comparable in 90-y-old singletons and long-lived siblings (9.45 nm and 9.42 nm, respectively), and the mean HDL particle size was significantly larger in 90-y-old singletons than partners (*p* < 10^−3^; Figure 3).

In order to investigate whether lipoprotein particle subclasses mark not only longevity but also health status, we assessed the overall health status in long-lived siblings and 90-y-old singletons using questionnaires about daily activities. A lower concentration of particularly small LDL particles was associated with better health in both long-lived siblings (*p* = 0.003) and the 90-y-old singletons (*p* = 0.007). This result is consistent with the findings on longevity. For total HDL particle concentration, however, these analyses revealed an opposite effect. Whereas long-lived siblings and 90-y-old singletons had non-significantly fewer HDL particles than younger controls, within these elderly groups fewer HDL particles were associated with poorer health (both *p* < 10^−4^).

Part of the variation in particle size and concentration may be attributable to differences in triglyceride levels, which can be measured routinely [9,10]. The differences found for LDL particle phenotypes between partners and offspring, however, did not appreciably change after adjustment for triglyceride levels (*p* = 0.028, *p* = 0.014, and *p* = 0.012 for size, total, and small particle concentration, respectively), indicating the added value of explicitly measuring particle size and concentration. The only significant difference between partners and offspring we observed for a HDL particle phenotype, namely among women for total HDL particle concentration, however, did not remain statistically significant after adjusting for triglyceride levels (*p* = 0.11). The differences for LDL particle phenotypes between partners and offspring also persisted after adjustment for triglyceride levels as well as classical LDL and HDL cholesterol levels (*p* = 0.038, *p* < 0.01, and *p* < 0.01 for size, total, and small particle concentration, respectively).

## Discussion

We employed a study design tailored to identify genetic and biochemical markers for human longevity and healthy ageing. We studied Dutch families consisting of long-lived siblings, their offspring, and partners of their offspring, who served as controls. Human longevity clustered in these families: parents, siblings, and, crucially, offspring of the recruited long-lived siblings have an on average 30% lower mortality rate than their birth cohort [4]. In the current study, we investigated whether lipoprotein particle profiles in blood are associated with human longevity. We found that the offspring of long-lived siblings had significantly larger and fewer LDL particles than their partners who may be considered matched by age and household environment. The contrast in both LDL particle phenotypes was even more prominent when partners were compared with long-lived siblings. These two associations were traced back to a single phenotype being a lower concentration of small LDL particles. Our study constitutes the first independent confirmation of findings previously described in long-lived families from the Ashkenazi Jewish founder population, namely, a significantly larger mean LDL particle size among offspring of long-lived individuals than their partners (0.2 nm larger for men and 0.5 nm for women, respectively) [8]. Moreover, it extends the association of LDL particle phenotypes with familial longevity to an outbred, European population. Also, we now identified small LDL particle concentration as the main culprit and showed that the influence of LDL particle phenotypes is independent of triglyceride levels, which are main determinants particularly of the LDL size [9,10] that was studied in the original report. Of note, we found significantly higher classical LDL-cholesterol levels among offspring than partners, but we did not observe similarly increased LDL-cholesterol levels among long-lived siblings. Although we currently do not have an explanation for this finding, it reinforces the notion that familial longevity is not associated with low LDL-cholesterol levels [8]. Our study thus underscores the added value of detailed measurements on lipid metabolism, such as lipoprotein particle concentrations, instead of measuring classical lipid levels only.

The relevance of LDL size and subclasses, however, may not be confined to familial longevity. The 90-y-old singletons from a population-based survey equally showed a larger mean LDL particle size. The great similarity in familial and sporadic human longevity suggests that both genetic and environmental factors may induce the beneficial LDL particle phenotype that marks the propensity for human longevity. Also, in both long-lived siblings and 90-y-old singletons a lower concentration of small LDL particles was associated with better overall health. Taken together, our data indicate that an under-representation of small LDL particles marks longevity and health in the general population and is associated with the propensity for longevity already in middle age. Future studies in cohorts of elderly individuals prospectively followed for a long period are warranted to explore the associations of small LDL particles in depth.

Longevity in long-lived Ashkenazi Jewish families was also marked by a larger mean HDL particle size [8]; however, we could not replicate this result. The single significant difference between partners and offspring we observed was for total HDL particle concentration among women, but correction for triglyceride levels abolished the significance of this association. Despite the absence of an association in this age-controlled comparison, larger and fewer HDL particles were found in the long-lived siblings as compared with their offspring and controls. Although this may be in line with a beneficial association of HDL particle phenotypes in old age, we found opposite associations of total HDL particle concentration with an indicator of overall health status in both nonagenarian groups. In Ashkenazi Jewish families, homozygosity of the I405V variant in the *CETP* gene was associated with a larger mean HDL size and its frequency significantly increased from controls, offspring to centenarians. In our study, however, this genotype was not associated with a larger mean HDL size nor did we and others [17] find a significant association with longevity (unpublished data). Our data are compatible with the interpretation that HDL particle phenotypes are a consequence of unknown factors associated with old age. The disparate outcomes between the studies may be due to differences in inclusion criteria, inclusion of confounders, and populations studied.

On the basis of the current and previous study [8], it may be speculated that small LDL particles are a candidate biomarker for the rate of biological ageing. Offspring of long-lived siblings with a familial propensity for human longevity express this marker already around the age of 60 y, long before the longevity phenotype becomes evident. It is, however, unclear whether small LDL particles play a causal role. Small LDL particles have been suggested to promote atherosclerosis because they are susceptible to oxidation and may easily accumulate in the arterial wall [18]. This hypothesis is supported by the finding that the concentration of small LDL particles predict the risk of cardiovascular disease [9], although large LDL particles have also been associated with coronary heart disease [18]. An intriguing aspect of our study is that long-lived men and women had a much more comparable LDL particle concentration than male and female controls with a mean age of 60 y. This finding might be associated with the convergence of the sex-specific cardiovascular mortality rates in old age. An alternative explanation for our finding is that small LDL particles are not causally involved but mark an adverse metabolic state that contributes to the future risk of disease or monitor early features of disease. The occurrence of smaller mean LDL size among type II diabetes patients has been well established since the early 1990s [19], and, recently, a higher concentration of small LDL particles as measured with NMR was shown to be strongly associated with lower insulin sensitivity [10]. The relationship with metabolic disease might reflect that small LDL particles mark mechanisms involved in the rate of biological ageing. Studies into the determinants of maximum lifespan in animal models have revealed pathways that may be important for human longevity. They indicated, for example, that genes from the insulin pathway regulate lifespan across species, simultaneously affecting stress response, fertility, and basic metabolic properties [20]. Lipid transport and storage may likewise constitute a central process in ageing and longevity [21].

In conclusion, we found evidence that a low concentration of small LDL particles marks human longevity already in middle age. Our study extends the finding that LDL particles are associated with human longevity in a genetically isolated population to an outbred European Caucasian population. It further indicated that this association is present not only among individuals with exceptional familial longevity but also in the population at large. Since long-lived individuals escape death from diverse common diseases, including cardiovascular diseases and cancer [6], it will be important to establish whether our finding reflects resistance to a specific disease or indicates longevity-associated pathways that have a broader effect on ageing, for example by regulating metabolism, stress resistance, or immune function.

## Supporting Information

Figure S1 LDL and HDL Particle Concentrations in Partners, Offspring, and Long-Lived Siblings according to Age Strata (A) LDL; (B) HDL. Error bars are standard errors. LDL particle concentration was positively associated with age (*p* = 0.028); HDL particle concentration was not (*p* = 0.74).
(5.1 MB TIF)

### Accession Numbers

The Online Mendelian Inheritance in Man (OMIM; http://www.ncbi.nlm.nih.gov/entrez/query.fcgi?db=OMIM) accession numbers for genes mentioned in the text are *APOB* (107730)*, APOE* (107741), and *CETP* (118470).

## Abbreviations

ADL: activities of daily living
HDL: high-density lipoprotein
LDL: low-density lipoprotein
